# Microcavity top-emission perovskite light-emitting diodes

**DOI:** 10.1038/s41377-020-0328-6

**Published:** 2020-05-22

**Authors:** Yanfeng Miao, Lu Cheng, Wei Zou, Lianghui Gu, Ju Zhang, Qiang Guo, Qiming Peng, Mengmeng Xu, Yarong He, Shuting Zhang, Yu Cao, Renzhi Li, Nana Wang, Wei Huang, Jianpu Wang

**Affiliations:** 1 Key Laboratory of Flexible Electronics (KLOFE) & Institute of Advanced Materials (IAM), Nanjing Tech University (NanjingTech), 30 South Puzhu Road, Nanjing, 211816 China; 2 Frontiers Science Center for Flexible Electronics (FSCFE), Shaanxi Institute of Flexible Electronics (SIFE) & Shaanxi Institute of Biomedical Materials and Engineering (SIBME), Northwestern Polytechnical University (NPU), 127 West Youyi Road, Xi’an, 710072 China

**Keywords:** Lasers, LEDs and light sources, Optical physics

## Abstract

Light-emitting diodes (LEDs) based on perovskites show great potential in lighting and display applications. However, although perovskite films with high photoluminescence quantum efficiencies are commonly achieved, the efficiencies of perovskite LEDs are largely limited by the low light out-coupling efficiency. Here, we show that high-efficiency perovskite LEDs with a high external quantum efficiency of 20.2% and an ultrahigh radiant exitance up to 114.9 mW cm^−2^ can be achieved by employing the microcavity effect to enhance light extraction. The enhanced microcavity effect and light out-coupling efficiency are confirmed by the study of angle-dependent emission profiles. Our results show that both the optical and electrical properties of the device need to be optimized to achieve high-performance perovskite LEDs.

Metal halide perovskites are becoming promising candidates for planar light-emitting diode (LED) applications^1–8^ due to their unique optoelectronic properties such as high photoluminescence quantum efficiency (PLQE)^9^ and good color purity^10^. During just 5 years, the external quantum efficiencies (EQEs) of perovskite light-emitting diodes (PeLEDs) have been enhanced from below 1% to over 20%^1,11–16^. Many efforts have been made to develop high-efficiency PeLEDs with good stability^17^, e.g., by employing multiple-quantum-well (MQW)-based perovskites^3,4,18^. However, although perovskite films with high PLQEs are commonly achieved, the efficiencies of PeLEDs are largely limited by the low light out-coupling efficiency. Here, we demonstrate high-efficiency perovskite LEDs by employing the microcavity effect to enhance light extraction. The enhancement of the light out-coupling efficiency is confirmed by comprehensive studies of angle-dependent emission profiles. Moreover, our results show that both the optical and electrical properties of the device need to be optimized to achieve high-performance PeLEDs.

For a PeLED, the EQE can be expressed as EQE = *η*_inj_ × *η*_PLQE_ × *η*_out._ Here, *η*_inj_ is the carrier capture efficiency, *η*_PLQE_ is the photoluminescence quantum efficiency (PLQE) of the perovskite film, and *η*_out_ is the light out-coupling efficiency of the device. Previous works have shown that excellent carrier capture efficiency can be achieved through optimization of the device structure^11,19^. To enhance the PLQE of an emitter, either suppressing the non-radiative recombination or promoting the radiative recombination can be viable solutions^7,20^. For MQW perovskites, it has been demonstrated that the non-radiative recombination can be manipulated by tuning the widths of the quantum wells, which can be controlled by changing the precursor solutions and process conditions^11,21^. This strategy is commonly used to achieve MQW perovskite films with high PLQEs up to 70%^11^. On the other hand, the radiative decay rate can be increased due to the Purcell effect^22^ when placing an emitter in a Fabry–Perot microcavity with an appropriate optical length^23^. Thus, we expect that the emission properties of an optimized MQW perovskite film can be further enhanced by using the microcavity effect. Moreover, since the enhanced emission is directed along the optical axis of the cavity^24^, a large proportion of the emission should be small-angle light. Therefore, the light out-coupling efficiency can also be enhanced, which is important for most types of LEDs^25^.

In the present work, a microcavity was formed by using a total-reflection Au bottom electrode and a semitransparent Au top electrode in a simple top-emission (TE) LED device structure. The length of the cavity was tuned by changing the thickness of the carrier transport layers. The structure of the TE-PeLEDs was optimized to be glass/Au (100 nm)/polyethylenimine ethoxylated-modified zinc oxide (ZnO, 37 nm)/MQW perovskite (approximately 35 nm)/poly(9,9-dioctyl-fluorene-co-N-(4-butylphenyl)diphenylamine) (TFB, 76 nm)/molybdenum oxide (MoO_3_, 7 nm)/Au (15 nm), as shown in Fig. 1a. The flat-band energy level diagram of the device is shown in Fig. S1. Figure 1b shows the current density–radiant exitance–voltage characteristics. The current density before device turn-on is ~1 mA cm^−2^, which is similar to that of high-performance PeLEDs in the literature^12,13,16^. After turn-on of the EL at a voltage of 1.6 V, the radiant exitance quickly rises and reaches a high value up to 114.9 mW cm^−2^ at 4.8 V. As shown in Fig. 1c, we achieved a high peak EQE of 20.2% at 3.7 V with a current density of 130 mA cm^−2^, which is maintained at 17.4% even under a high current density of 400 mA cm^−2^. To verify the reproducibility of the performance, 61 devices were fabricated. The EQE histogram shows an average EQE of 17.5% with a small relative standard deviation of 8.2% (Fig. 1d). In comparison, the best-performance bottom-emission (BE) PeLEDs based on the same MQW perovskite show a peak EQE of 14.5%. This is consistent with the finite-difference time-domain-based optical simulations, which indicate that due to the presence of the microcavity effect, the light out-coupling efficiency of the TE-PeLEDs (~30%) is larger than that of the bottom-emission devices (~20%) at approximately 800 nm (Fig. S10). The detailed optimization of the MQW perovskite films and the performance of the BE-PeLEDs are shown in Figs. S2–S4.

**Fig. 1:**
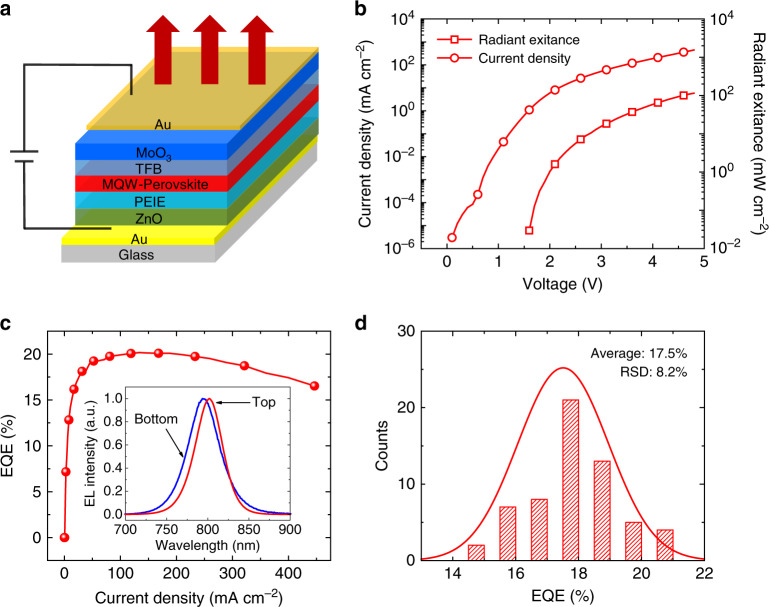
Device structure and optoelectronic characteristics of the top-emission PeLEDs. **a** Structure of the TE-PeLEDs. A thick gold film was used as the total-reflection bottom electrode, and a thin gold film was used as the semitransparent top electrode, forming a Fabry–Perot microcavity. The light is emitted through the top electrode. **b** Dependence of the current density and radiant exitance on the driving voltage. A high radiant exitance of 114.9 mW cm^−2^ was achieved at a voltage of 4.8 V. **c** EQE versus current density. A high EQE of 20.2% was achieved at 3.7 V with a current density of 130 mA cm^−2^. The inset shows a comparison of the EL spectra for the TE- and BE-PeLEDs. **d** Histogram of peak EQEs for the TE-PeLEDs. The statistics of 61 devices show an average EQE of 17.5% with a relative standard deviation of 8.2%.

The microcavity resonance effect of our high-efficiency TE-PeLEDs can be confirmed by the angular-dependent emission profiles. The full-width at half-maximum (FWHM) of the electroluminescence (EL) spectrum of the TE-PeLED in the forward direction (39 nm) is narrower than that of the conventional BE-PeLED based on the same emitter (46 nm) (the inset in Fig. 1c). Moreover, the EL spectrum of the TE-PeLED gradually blueshifts with increasing viewing angle, as shown in Figs. 2a and S5b. We believe that the angle-dependent EL spectrum originates from the microcavity effect, as confirmed by the calculations based on the Fabry–Perot equation (see “Methods”)^26,27^. When the viewing angle is larger than 40°, the EL intensity of the TE-PeLED decreases much faster than that of the BE-PeLED (Fig. S5a, b), indicating that the proportion of small-angle light to total light is larger in the TE-PeLED than in the BE-PeLED. This is clearly verified by the angular-dependent profiles of the emission at different wavelengths (Fig. 2b, d). In contrast, the BE-PeLED exhibits a Lambertian emission profile (Fig. 2d)^28^, and no spectrum shift was observed with changing viewing angle (Figs. 2c and S5a). In addition, we measured the PL lifetimes when the perovskite was inside and outside the cavity (Fig. S8). The PL lifetime decreased when the perovskite was inside the microcavity, consistent with the microcavity resonance effect^27,29^.

**Fig. 2:**
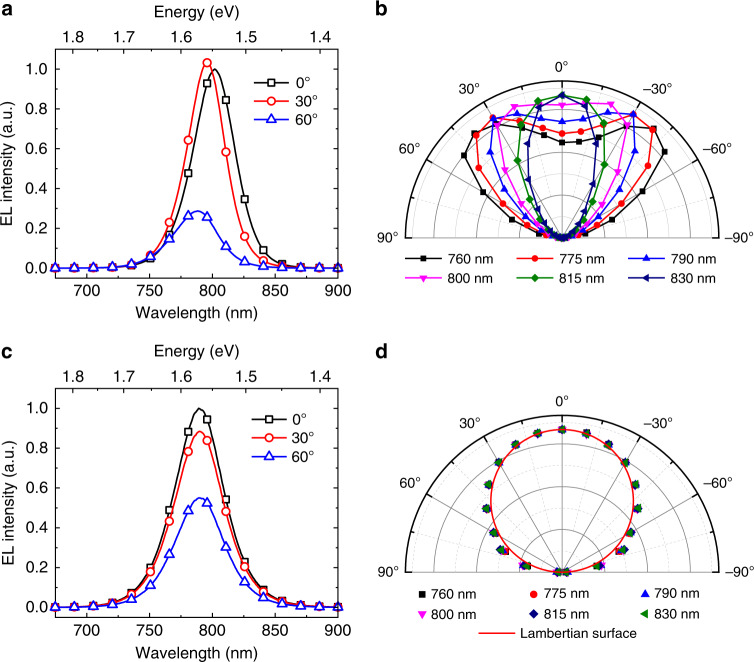
Angular-dependent emission profiles of the PeLEDs. **a** EL spectra of the TE-PeLED at 0, 30, and 60°. The EL is blueshifted with increasing viewing angle. **b** Angular-dependent profiles of the emission of the TE-PeLED at different wavelengths, which clearly show that most of the EL is small-angle light. **c** EL spectra of the BE-PeLED at 0, 30, and 60°. The EL spectra remain unchanged in different directions. **d** Angular-dependent profiles of the emission of the BE-PeLED at different wavelengths, which follow a Lambertian profile.

We then explore how to make rational use of the microcavity effect to maximize the efficiency in the PeLEDs. First, the impact of the cavity length on the device efficiency was studied. TE-PeLEDs with different cavity lengths were fabricated by changing the thicknesses of the ZnO and TFB layers. As shown in Figs. 3a and S6, when the cavity length is such that the resonant wavelength of the cavity matches the emission wavelength of the film, we can achieve the best device performance. Here, we mention that both the optical and electrical properties should be affected when the layer thickness is changed. Therefore, the improvement of the device efficiency may also be partly due to the improvement of the electrical properties. This entangled optical–electrical effect will be discussed below. Second, we investigated the effects of the thickness of the semitransparent electrode on the device performance. When the thickness of the top electrode is increased, on the one hand, the reflectivity will be increased, leading to an enhanced microcavity effect; on the other hand, the transmissivity will be decreased, and thus, the light out-coupling efficiency should be reduced. Figures 3b and S7 show the performances of devices with top electrodes of different thicknesses. The FWHM of the emission decreases (from 42 to 35 nm) with increasing thickness of the top electrode (from 8 to 30 nm) (Fig. S7a), demonstrating the enhanced microcavity effect. However, the TE-PeLED with a 15 nm top electrode displays the highest EQE among the three devices (Fig. 3b), revealing the importance of selecting a top electrode of appropriate thickness. Finally, we calculated the device efficiencies when changing the position of the emission zone in the cavity (see “Methods”)^29^. For our half-wave microcavity PeLEDs, the intensity of the emission reaches a maximum when the emission zone is near the center of the cavity (Fig. 3c). The cause of the deviation from the center is that our bottom Au electrode is not a perfect metal mirror, and thus, the phase shift is not *π*.

**Fig. 3:**
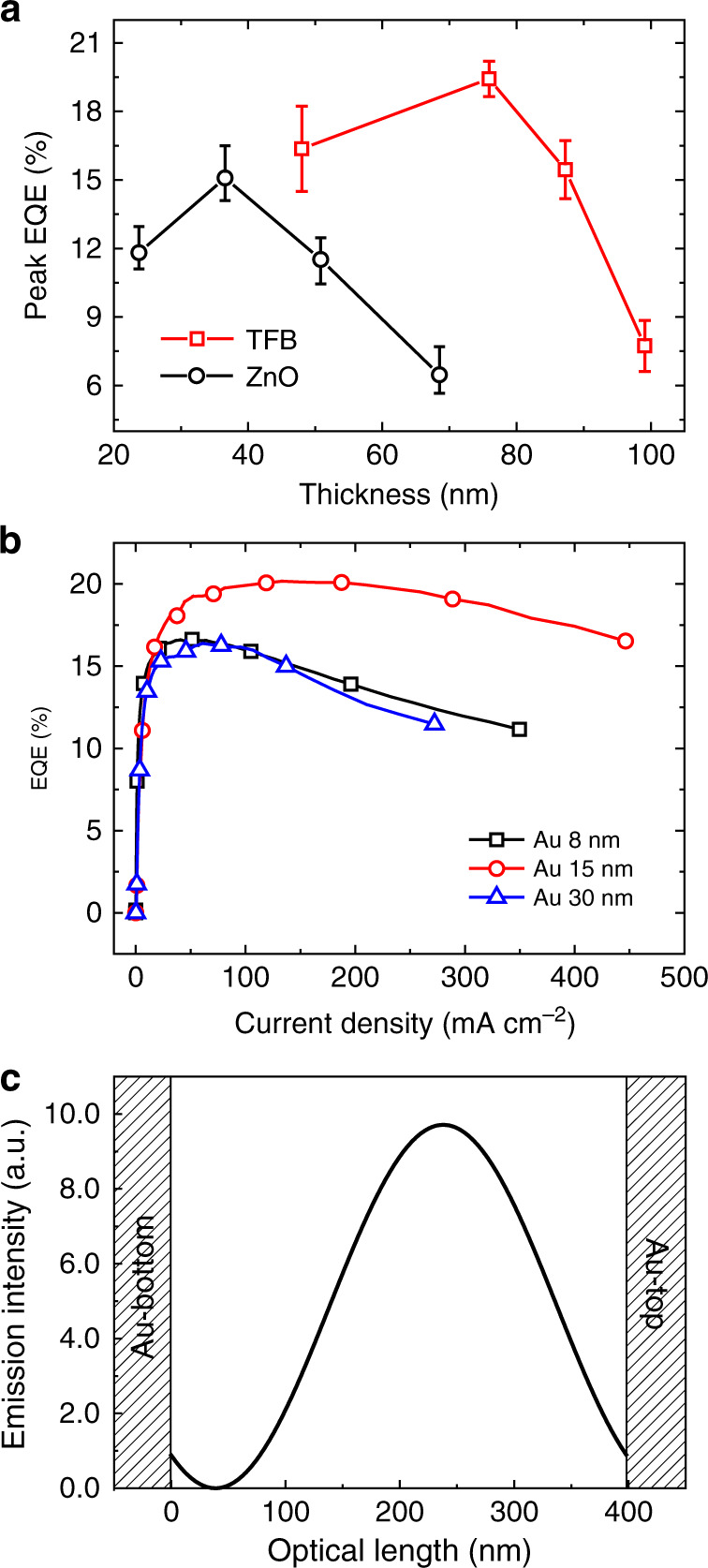
Impacts of the cavity length, top electrode thickness, and emitter position on the performance of the TE-PeLEDs. **a** Peak EQEs of the devices with carrier transport layers of different thicknesses. The EQE reaches a maximum when the cavity length is suitable such that the resonant wavelength of the cavity matches the emission wavelength of the emitter. **b** EQE versus current density for three top-emission PeLEDs. The device with a 15 nm top electrode displays the highest peak EQE (20.2%), which is higher than those of the devices with 8 nm (16.6%) and 30 nm (16.4%) top electrodes. **c** Emission intensity as a function of the optical length between the emission zone and the bottom electrode. The emission intensity reaches a maximum when the emission zone is near the center of the cavity.

The above analyses suggest that to achieve a highly efficient microcavity TE-PeLED, the optical and electrical properties of the devices should be comprehensively considered. For the optical properties: (1) According to the microcavity resonance principle, the resonant wavelength should match the emission of the materials, enabling the emission profile to be advantageous for light extraction and enhancing the radiative decay rate. (2) The thickness of the semitransparent top electrode should be suitable, ensuring a sufficiently strong microcavity effect while keeping the light absorption by the top electrode sufficiently small. (3) The emitter should be placed in the resonant node, guaranteeing constructive interference. For the electrical properties: (1) The carrier transport layers should have an appropriate thickness, ensuring efficient carrier transport while avoiding quenching of the excited state by the electrodes. (2) To guarantee the charge balance, the thickness of the ZnO should not be greater than that of the TFB since they have similar mobilities^30,31^ and the charge recombination zone in the device is near the emitting layer/hole transport layer interface^3^. However, it is difficult to fulfill all the optical requirements while achieving balanced carrier injection/transport. Thus, to achieve high-efficiency TE-PeLEDs, we should make a trade-off between the optical and electrical properties. This can account for the deviations that occurred in our TE-PeLEDs. That is, (1) the smallest FWHM (35.4 nm) was obtained at a viewing angle of 30° and not in the normal direction (Fig. S5c), and (2) the EL peak of the TE-PeLED was redshifted by approximately 7 nm compared with that of the BE-PeLED (inset in Fig. 1c). Both phenomena indicate that the resonant wavelength of the TE-PeLED is longer than the emission peak of the film. However, this device exhibits the highest EQE among many devices with carrier transport layers of different thicknesses (and different cavity lengths), probably because the device is just at the balance point between the optical and electrical characteristics.

In summary, we have investigated the optoelectronic properties of microcavity top-emission PeLEDs. For planar LEDs, such as organic light-emitting diodes and PeLEDs, light trapping is the major factor of efficiency loss^32,33^, and it can be more serious for PeLEDs because of the higher refractive indices of perovskites than those of organic semiconductors. In this work, by employing a top-emission device structure with the microcavity effect, we show that the light extraction can be largely improved, and a high peak EQE of 20.2% was achieved. Importantly, the demonstration of the microcavity effect in perovskite LED devices is a meaningful step towards electrically pumped perovskite lasers.

## Methods

### Synthesis and material preparation

Colloidal ZnO nanocrystals were synthesized by a solution-precipitation process^2^. The perovskite precursor solution was prepared from N-methylacridinium iodide (NMAI), formamidinium iodide, and PbI_2_ with different molar ratios (from 2:1.9:2 to 1.3:1.9:2) in dimethylformamide (9 wt%) and stirred overnight at room temperature in a nitrogen-filled glove box. The NMAI was synthesized similar to the previously reported method^3^.

### Device fabrication

A similar description of the device fabrication process can be found elsewhere for BE-LEDs^3^. For TE-PeLEDs, a bottom Au electrode of 100 nm was deposited onto a glass substrate using a thermal evaporation system through a shadow mask under a pressure of 6 × 10^−7^ torr. The device area was 3 mm^2^ as defined by the overlapping area of the bottom and top Au electrodes.

### Characterization

A detailed description of the device performance characterization can be found elsewhere^3^. The angular dependence of the emission spectra was measured by using a Thorlabs F280SMA fibre collimator coupled with a QE65 Pro spectrometer at a fixed distance of 200 mm from the EL device.

Ellipsometry measurements were carried out with a Horiba Jobin Yvon UVISEL iHR320 ellipsometer under an incident angle of 60° for photon energies between 0.6 and 6 eV with a 10 meV increment.

### Calculations

#### Resonant wavelength of the microcavity

The resonant wavelengths of our TE-PeLEDs were calculated by using the Fabry–Perot equation^27^:

1 $${\sum} {{\it{n}}_{i}d_{i}{\mathrm{cos}}{\uptheta}_{i} + \left| {\frac{{\varphi _{\mathrm{top}}}}{{4\pi }}\lambda } \right| + \left| {\frac{{\varphi _{\mathrm{bot}}}}{{4\pi }}\lambda } \right|{\mathrm{ = }}\frac{m}{2}\lambda \quad \left( {m = 1,2, \cdots } \right)}$$

where *λ* is the resonant wavelength of the microcavity; *n*_*i*_ and *d*_*i*_ are the refractive index and thickness of the *i*th layer between the two electrodes; and *θ*_*i*_ is the light propagation direction in the *i*th layer. *φ*_top_ and *φ*_bot_ are the phase shifts at the top- and bottom-electrode contacts, respectively, which can be calculated by^26,27^:

2 $$\varphi _m = {\mathrm{arctan}}\left[ {\frac{{{\it{Im}}\left( r \right)}}{{{\it{Re}}\left( r \right)}}} \right] = {\mathrm{arctan}}\left[ {\frac{{2n_0k_1}}{{n_0^2 - n_1^2 - k_1^2}}} \right]$$

where *Im*(*r*) and *Re*(*r*) are the imaginary and real parts of the reflection coefficient of the metal electrodes, respectively. *n*_0_ is the refraction coefficient of the incident layer in contact with the metal electrodes, and *n*_1_ and *k*_1_ are the refractive index and extinction coefficient of the metal electrodes, respectively.

For the calculation, we measured the refractive index (*n*) and extinction coefficient (*k*) of the layers in the devices (Fig. S9). The calculated resonant wavelengths in different directions are shown in Fig. S5d. We note that the calculated resonant wavelengths are shorter than the experimental values, probably due to instrument errors in the measurements of *n*/*k* and the thickness of the layers. Nonetheless, it is apparent that the resonant wavelength becomes shorter with increasing direction angle.

#### Emission intensity with changing emitter position

The emission intensity of the device was calculated by^29^:

3 $${{I}}\left( \lambda \right) = \frac{{T_{\mathrm{top}}\left[ {1 + R_{\mathrm{bot}} + 2\sqrt {R_{\mathrm{bot}}} {\cos} \left( -{\varphi _{{\boldsymbol{\mathrm{bot}}}} + \frac{{4\pi nd}}{\lambda }} \right)} \right]}}{{1 + R_{\mathrm{bot}}R_{\mathrm{top}} - 2\sqrt {R_{\mathrm{bot}}R_{\mathrm{top}}} {\cos} \left( {\frac{{4\pi L_{\mathrm{cav}}}}{\lambda }} \right)}}I_{0}\left( \lambda \right)$$

where *T*_top_ (*T*_bot_) and *R*_top_ (*R*_bot_) are the transmittance and reflectivity of the top (bottom) electrode, *φ*_bot_ is the phase shift in the bottom electrode, and *d* is the optical length from the emission zone position to the bottom electrode. *I*_0_(*λ*) is the emission intensity without the microcavity effect.

## Supplementary information

Supplemental Material
