# Supplementary material for: Microcavity top-emission perovskite light-emitting diodes
Source: Light Sci Appl. 2020 May 22;9:89. doi: 10.1038/s41377-020-0328-6 (PMC7242415; doi:10.1038/s41377-020-0328-6)
Supplement: Supplementary file 1 — Supplemental Material [file 41377_2020_328_MOESM1_ESM.pdf]

Supplementary Information

for

Microcavity Top-emission Perovskite Light-emitting Diodes

Yanfeng Miao[1†], Lu Cheng[1†], Wei Zou[1†], Lianghui Gu[1], Ju Zhang[1], Qiang Guo[1], Qiming Peng[1]*, Mengmeng Xu[1], Yarong He[1], Shuting Zhang[1], Yu Cao[1], Renzhi Li[1], Nana Wang[1], Wei Huang[1,2]* and Jianpu Wang[1]*

[1]*Key Laboratory of Flexible Electronics (KLOFE) & Institute of Advanced Materials (IAM), Nanjing Tech University (NanjingTech), 30 South Puzhu Road, Nanjing 211816, China.*

[2]*Frontiers Science Center for Flexible Electronics (FSCFE), Shaanxi Institute of Flexible Electronics (SIFE) & Shaanxi Institute of Biomedical Materials and Engineering (SIBME), Northwestern Polytechnical University (NPU), 127 West Youyi Road, Xi'an 710072, China.*

[†]*These authors contributed equally to this work.*

**Correspondence to: iamqmpeng@njtech.edu.cn (Qiming Peng); iamwhuang@nwpu.edu.cn (Wei Huang); iamjpwang@njtech.edu.cn (Jianpu Wang)*

1. The flat-band energy level diagram of the TE-PeLEDs.

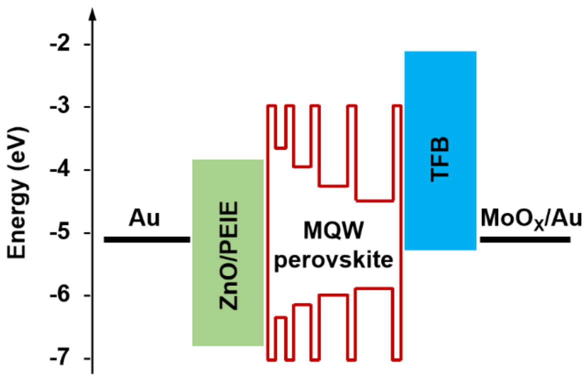

Figure S1. The flat-band energy level diagram of the TE-PeLEDs. There is an energetic offset of 1.3 eV between the CBM of ZnO and the work function of Au, and an energy barrier of 0.7 eV between the VBM of perovskite and the HOMO of TFB.

2. Optimization of the MQW-perovskite films.

First of all, perovskite film with high PLQE is important for achieving high device efficiency. To gain high-PLQE, different MQW-perovskite films were formed by tuning the molar ratio of 1-naphthylmethylamine iodide (NMAI), formamidinium iodide (FAI), and PbI_2 in precursor solutions. The emission peaks at short-wavelength region revealed the formation of MQWs (Figure S2b). Though all the films exhibit similar smooth and pin-hole free morphologies (Figure S2a), the PL peaks of the MQW films exhibit slight red-shifts with decreasing the ratio of NMAI, revealing the redistribution of QWs with different widths[1,2]. Figure S3 shows the excitation intensity-dependent PLQEs of different films. With decreasing the ratio of NMAI from 2.0 to 1.4, the PLQEs become higher and rise faster, indicating the decreasing of trap density, which is in accordance with the decreased yellow phase of δ-$FAPbI_3$ (~12 ° and ~26 °) (Figure S2c).[3] However, further decrease of the molar ratio of NMAI would resulted in a decreased PLQE and rising rate, which can be attributed to the increased disorder of the perovskite film, as confirmed by the enhanced XRD peak of (123) crystal plane of α-$FAPbI_3$ at 31.5 ° (Figure S2c).[4] The best film shows a very high PLQE up to 78% when the molar ratio of NMAI, FAI and PbI_2 is tuned as 1.4:1.9:2.

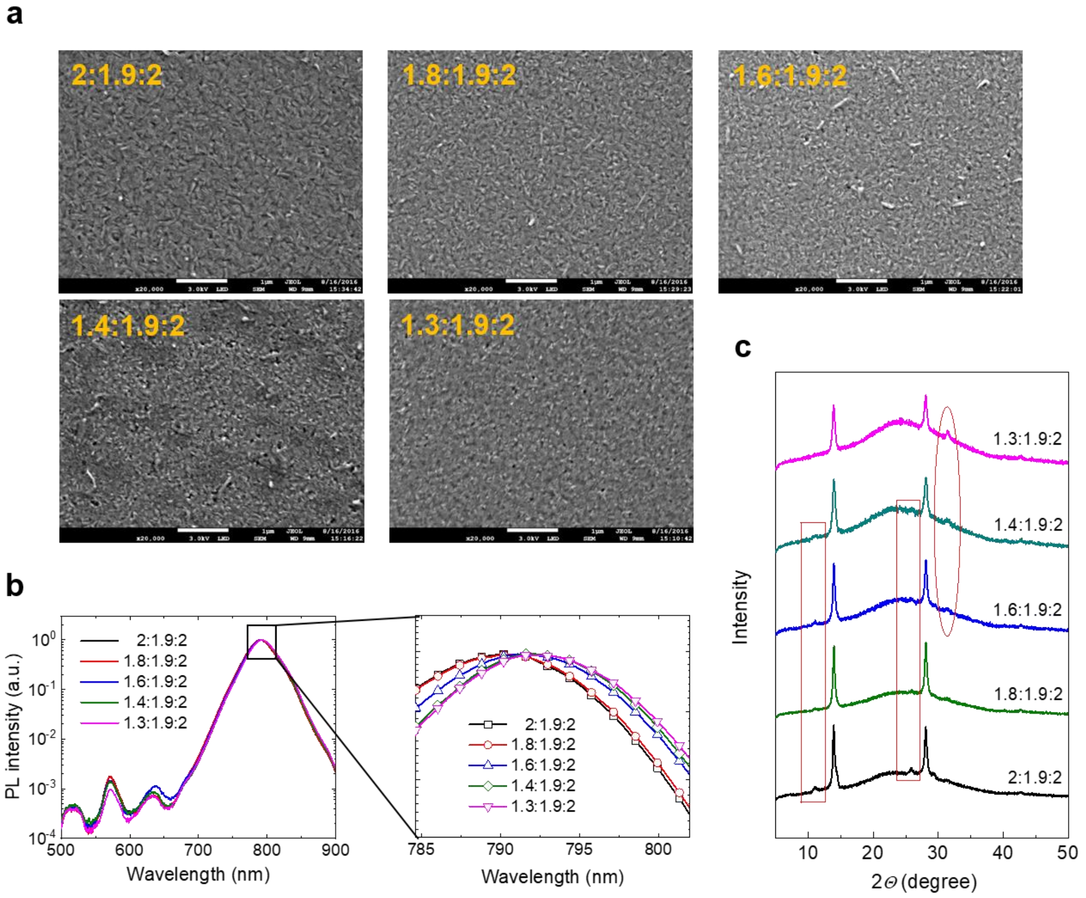

Figure S2. Characteristics of the MQW-perovskite films. **a**, SEM images. The films formed by NMAI, FAI, and PbI_2 of different molar ratio exhibit similar morphology. They are all smooth and pin-hole free. **b**, PL spectra of the films. The emission peaks at short-wavelengths reveal the formation of MQWs. The PL peaks exhibit slight red-shift with decreasing the ratio of NMAI, revealing the redistribution of QWs with different widths. **c**, XRD spectra. With decreasing the molar ratio of NMAI, yellow phase of δ-$FAPbI_3$ (~12° and ~26°) gradually diminished, but diffraction peak of the (123) crystal plane of α-$FAPbI_3$ at 31.5° gradually increased.

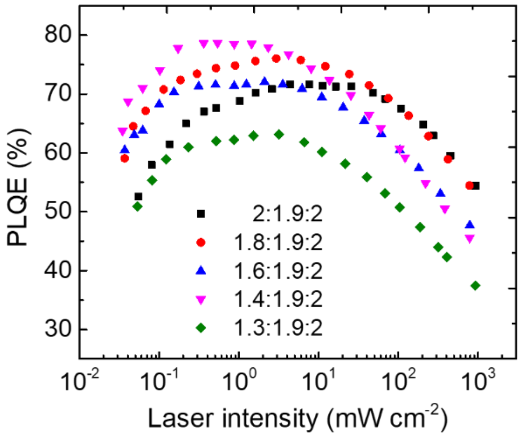

Figure S3. The excitation intensity-dependent PLQEs of different films formed by tuning the molar ratio of NMAI, FAI, and PbI_2 in the precursor solution. The 1.4:1.9:2-film exhibits a high peak PLQE up to 78%.

3. Device performance of the BE-PeLEDs.

BE-PeLEDs with the structure of indium tin oxide (ITO)/PEIE-modified ZnO (20 nm)/perovskite (30 nm)/TFB (40 nm)/MoO_3 (7 nm)/Au (60 nm) were fabricated. The optoelectronic performance of the BE-PeLEDs using different MQW-perovskite films are shown in Figure S4. The device using 1.4:1.9:2-film as the emitter show the highest peak EQE of 14.5% at 2.0 V with a current density of 10.6 mA cm^{-2} (Figure S4c). The average peak EQE of 63 devices is 12.8% with a relative standard deviation of 6.4% (Figure S4d), showing good reproducibility of the device performance.

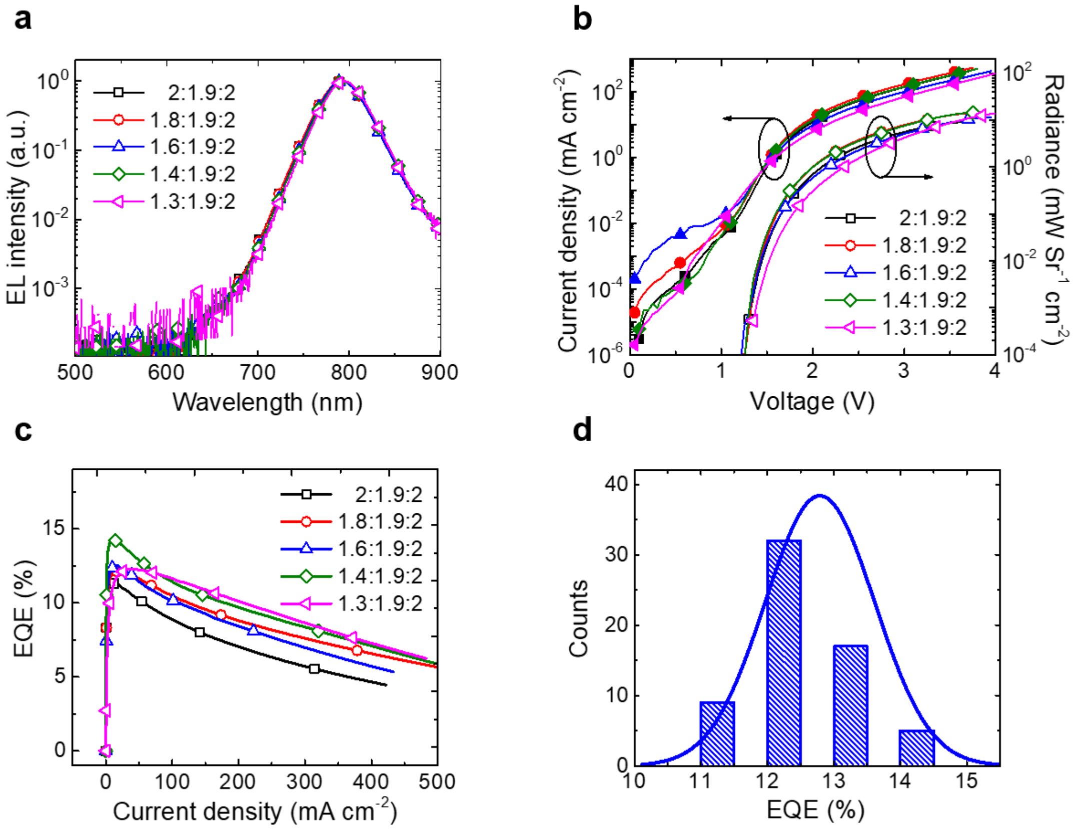

Figure S4. **a**, EL spectra of BE-PeLEDs based on the different films. **b**, Current density-radiance-voltage characteristics of the devices. **c**, EQE versus current density. A high peak EQE of 14.5% is achieved when the 1.4:1.9:2 MQW-film was used as the emitter. **d**, Histogram of peak EQEs for 63 BE-PeLEDs with1.4:1.9:2-film.

4. Device performance of the TE-PeLEDs.

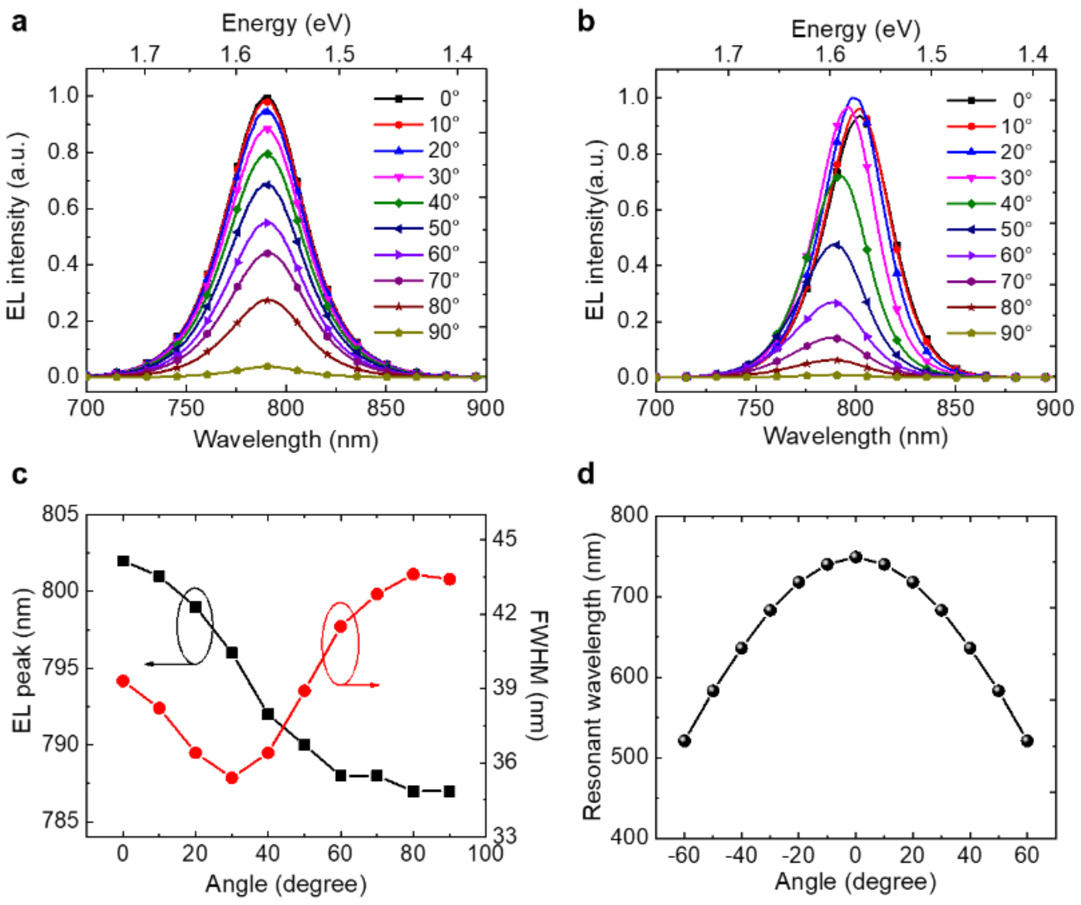

Figure S5. a, EL spectra of the BE-PeLEDs at different directions. Emissions in all viewing angles show an identical peak wavelength and FWHM. **b**, EL spectra of the TE-PeLEDs at different directions. The EL is blue-shifted when increasing the viewing angle. The EL intensity decreases faster than that of the BE-PeLEDs when the viewing angle is larger than 40 °. **c**, Dependence of Peak-wavelength and FWHM of the ELs of the TE-PeLEDs on the viewing angle. **d**, Calculated resonant wavelengths of our TE-PeLED at different directions.

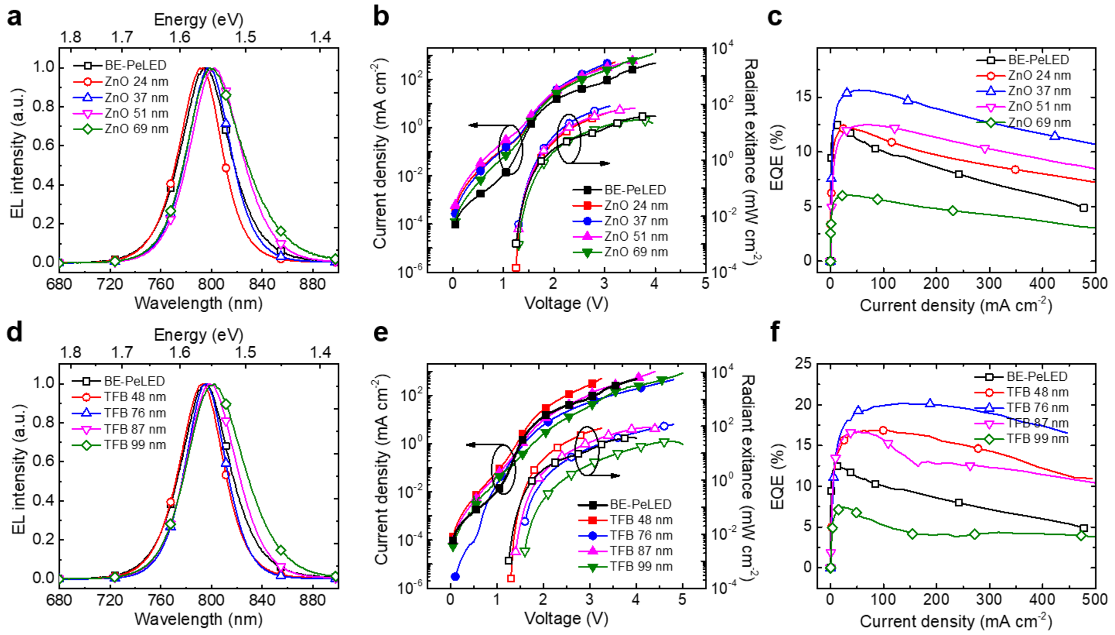

Figure S6. a-c, The EL spectra (of the total EL from all directions), the current density-radiance exitance-voltage characteristics, and the EQEs for TE-PeLEDs with different ZnO layers, respectively. Here the thickness of TFB was 48 nm. **d-f**, The EL spectra (of the total EL from all directions), the current density-radiance exitance-voltage characteristics, and the EQEs for TE-PeLEDs with different TFB layers, respectively. Here the thickness of ZnO was 37 nm.

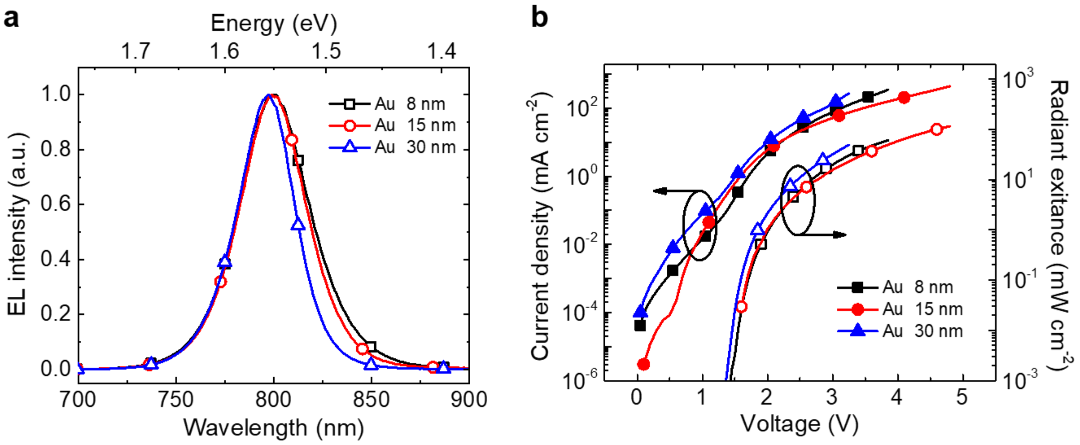

Figure S7. a, EL spectra of TE-PeLEDs with different top-electrode thicknesses. The FWHM of the emission decreases (from 42 nm to 35 nm) with increasing the thickness of the top-electrode (from 8 nm to 30 nm), revealing an enhanced micro-cavity resonant effect. **b**, Current density-radiance exitance-voltage characteristics. The device with top-electrode of 15 nm shows the best performance.

5. Transient PLs of the perovskite inside and outside the cavity.

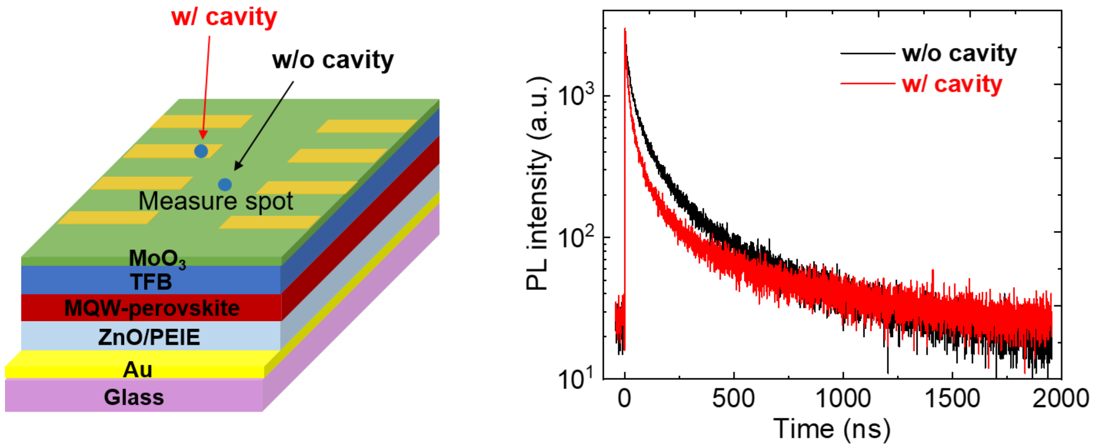

Figure S8. PL lifetimes of the perovskite film with and without the cavity under excitations of identical intensity.

6. Film characterization.

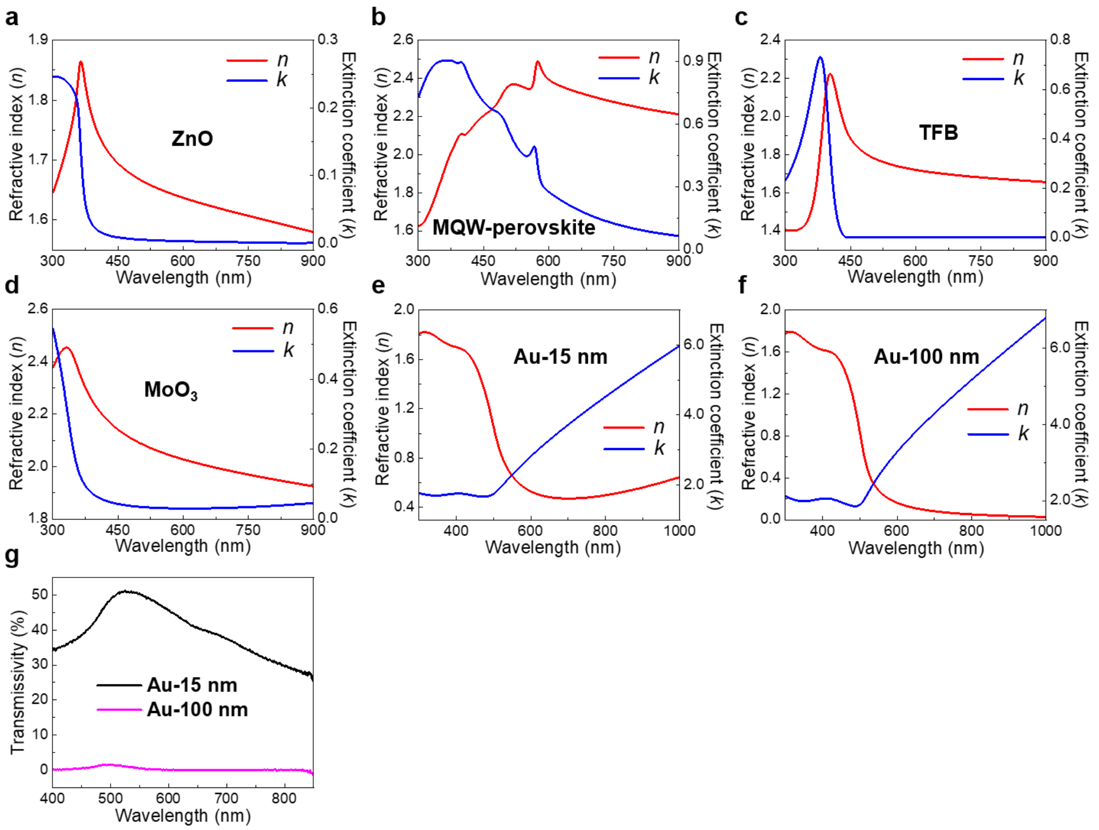

Figure S9. a-f, Refractive index (*n*) and extinction coefficient (*k*) of the layers of ZnO, MQW-perovskite, TFB and MoO_3, Au-15 nm and Au-100 nm, respectively. **g**, Transmissivity of Au layers with different thicknesses.

7. FDTD simulations.

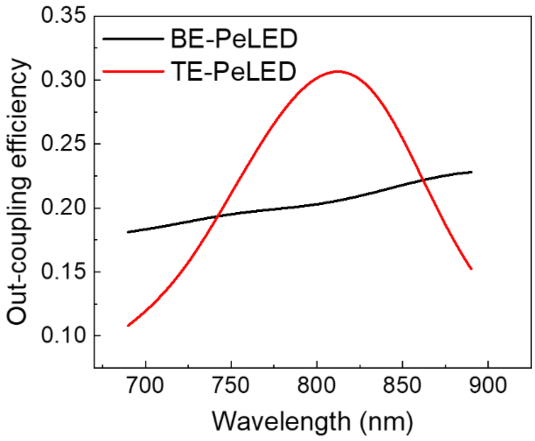

Figure S10. The light out-coupling efficiencies of the TE- and BE-PeLEDs for different wavelength emissions, calculated from the finite-difference-time-domain (FDTD) based optical simulations. The simulation details are shown in our previous work.[5]

References

1. Zou, W. *et al.* Minimising efficiency roll-off in high-brightness perovskite light-emitting diodes. Nat. Commun. **9**, 608 (2018).
2. Yang, X. *et al.* Efficient green light-emitting diodes based on quasi-two-dimensional composition and phase engineered perovskite with surface passivation. Nat. Commun. **9**, 570 (2018).
3. Jeon, N. J. *et al.* Compositional engineering of perovskite materials for high-performance solar cells. Nature **517**, 476-480 (2015).
4. Yang, W. S. *et al.* High-performance photovoltaic perovskite layers fabricated through intramolecular exchange. Science **348**, 1234-1237 (2015).
5. Cao, Y. *et al.* Perovskite light-emitting diodes based on spontaneously formed submicrometre-scale structures. Nature **562**, 249-253 (2018).
